# Transcriptome analysis reveals the molecular mechanisms of response to an emergent yellow-flower disease in green Chinese prickly ash (*Zanthoxylum schinifolium*)

**DOI:** 10.1038/s41598-021-98427-5

**Published:** 2021-09-23

**Authors:** Fan Xu, Qian Meng, Xiaodong Suo, Yonghong Xie, Yueqing Cheng, Ming Luo

**Affiliations:** 1 Key Laboratory of Biotechnology and Crop Quality Improvement of Ministry of Agriculture, Biotechnology Research Center, Southwest University, Chongqing, China; 2 Fruit Research Institute of Chongqing Academy of Agricultural Sciences, Chongqing, China

**Keywords:** Molecular biology, Plant sciences

## Abstract

Chinese prickly ash (*Zanthoxylum*) is extensively used as spice and traditional medicine in eastern Asian countries. Recently, an emergent yellow-flower disease (YFD) break out in green Chinese prickly ash (*Zanthoxylum schinifolium*, Qinghuajiao in Chinese) at Chongqing municipality, and then leads to a sharp reduction in the yield of Qinghuajiao, and thus results in great economic losses for farmers. To address the molecular response for the emergent YFD of Qinghuajiao, we analyzed the transcriptome of 12 samples including the leaves and inflorescences of asymptomatic and symptomatic plants from three different towns at Chongqing by high-throughput RNA-Seq technique. A total of 126,550 genes and 229,643 transcripts were obtained, and 21,054 unigenes were expressed in all 12 samples. There were 56 and 164 different expressed genes (DEGs) for the AL_vs_SL (asymptomatic leaf vs symptomatic leaf) and AF_vs_SF (asymptomatic flower vs symptomatic flower) groups, respectively. The results of KEGG analysis showed that the “phenylpropanoid biosynthesis” pathway that related to plant–pathogen interaction were found in AL_vs_SL and AF_vs_SF groups, and the “Plant–pathogen interaction” found in AF_vs_SF group, implying that this Qinghuajiao YFD might cause by plant pathogen. Interestingly, we detected 33 common unigenes for the 2 groups, and almost these unigenes were up-regulated in the symptomatic plants. Moreover, most of which were homologs to virus RNA, the components of viruses, implying that this YFD was related to virus. Our results provided a primary molecular basis for the prevention and treatment of YFD of Qinghuajiao trees.

## Introduction

Chinese prickly ash (also known as Huajiao in China) belongs to the Zanthoxylum genus of the Rutaceae family, and is a deciduous shrub native to East Asian countries^1,2^. According to its peel color, Huajiao is divided into red Chinese prickly ash and green Chinese prickly ash (also named as Qinghuajiao in Chinese). The fruits of Huajiao trees are largely used as a popular condiment in traditional cooking and medicine with a long history in China, which makes Chinese prickly ash an important economically fruit tree^3–6^. In particular, green Chinese prickly ash (*Zanthoxylum schinifolium*) is one of the most important commercial crops in Chongqing municipality. For instance, in 2018, approximately 530,000 acres of this variety were planted in Jiangjin district with a half billion dollars sale value. However, a yellow-flower disease broke out in Qinghuajiao at Chongqing in recent years, the infected trees showed phenotypes with pistil degeneration and stamen enlargement. Given that the normal green prickly ash was parthenogenesis, the infected plants could not bear fruit. On the other hand, with the growth of infected time, the leaves of diseased trees curled, and the root necrosis, which accelerated the death of Qinghuajiao trees, and then caused a serious impact on the production of Qinghuajiao, and thus resulted in huge economic losses.

Recently, with the rapid development of high-throughput sequencing technology, the study of transcriptomics has achieved greatly progresses. Transcriptome refers to the complete set of transcripts of an organism, a specific tissue, and a cell at a specific developmental stage or under a specific physiological condition^7^. Unlike a relatively stable genome, transcriptome alters with the change of biological conditions, stages of development, and external environmental factors. High-throughput transcriptome sequencing (high-through-put RNA-seq) is a powerful method to analyze the relationship between phenotype and genotype. It can accurately determine the gene expression level, differential splicing and specific expression of transcription products under specific conditions, so as to better understand the growth and development of cells and the underlying pathways and molecular mechanisms of disease progression^8,9^. The application of high-throughput RNA-Seq technology has promoted the study of plant–pathogen interactions with relatively complex genomes. Gray leaf spot (GLS), is one of the most impactful diseases in maize. Two maize cultivars ‘Yayu889’ (resistant) and ‘Zhenghong532’ (susceptible) were challenged with the GLS disease. The RNA-Seq results showed that there were 4666, 1733, and 1166 differentially expressed genes (DEGs) for ‘Yayu889’ cultivar, and 4713, 881, and 722 DEGs for ‘Zhenghong532’ cultivar after 8-, 10-, and 12-day infection, respectively, and which were mainly enriched in response to salicylic acid, protein phosphorylation, REDOX process, and carotenoid biosynthesis, and were especially active in the resistant cultivar^10^. Stripe rust is an important fungal foliar disease of wheat. Zhang et al. reported the transcriptome of wheat that infected by the powdery mildew E09 and stripe rust Cry31, and the results showed that Seven KEGG pathways were identified in response to the infection of powdery mildew and four pathways in response to the infection of stripe rust^11^. The RNA-Seq results of stripe rust infected wheat showed that the genes closely related to innate immunity of plants, such as biosynthesis and response genes related to plant stress hormones salicylic acid, jasmonic acid, ethylene and abscisic acid, were all significantly altered after strip rust infection. Meanwhile, genes encoding proteins with antimicrobial properties were found to be significantly enriched after the infection, including disease-related proteins, chitinases and cysteine-rich repeat proteins^12^.

Given that RNA-Seq does not require a genome sequence^7,13^, it allows us to define the global gene expression profiles for YFD infected Qinghuajiao trees. Here, we analyzed the transcriptome of 4 groups, 12 samples from three towns at Chongqing with severe disease by high-throughput RNA-Seq techniques. We found 56 and 164 different expressed genes (DEGs) for the leaf and flower samples of diseased trees. The results of KEGG pathway analysis showed that the DEGs for the diseased trees were involved in phenylpropanoid biosynthesis and plant–pathogen interaction pathways. In addition, we found 33 DEGs were shared by all samples from diseased trees, and most of these common genes were up-regulated in diseased trees and were highly homologous to the virus RNA, the components of viruses. These genes might be closely related to the YFD of Qinghuajiao. Our results provides an effective way for rapidly solving the outbreak diseases in agriculture.

## Results

### Phenotypes of YFD Qinghuajiao trees

**Figure 1:**
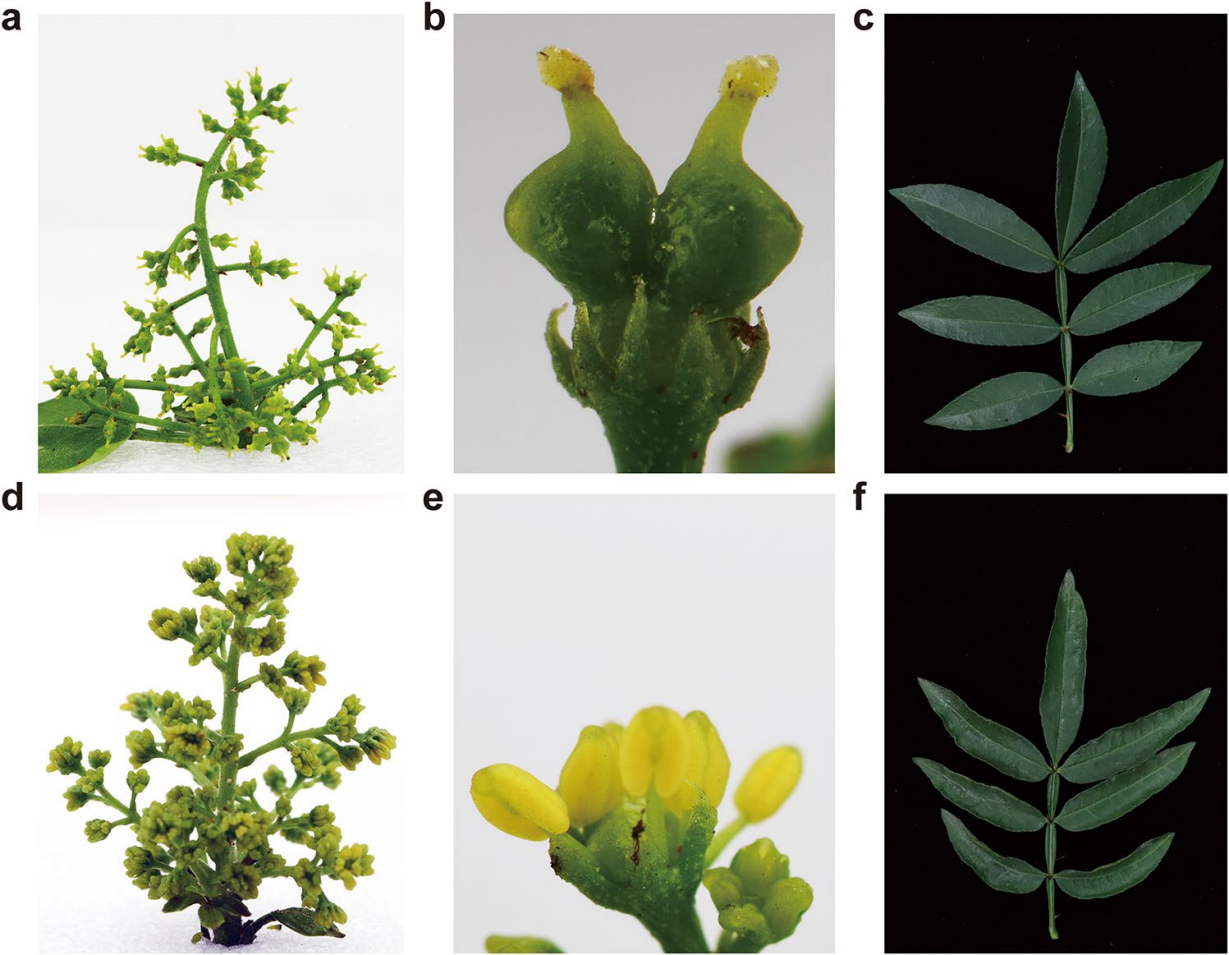
Phenotypes of symptomatic Qinghuajiao trees. (**a**–**c**) Normal top inflorescence, flower (from **a**), and the antepenultimate leaf of healthy 5-year old Qinghuajiao trees grown in the experimental field of Fruit Research Institute of Chongqing Academy of Agricultural Sciences, at Diaojia town under natural conditions. (**d**–**f**) Yellow top inflorescence, flower (from **d**), and curled antepenultimate leaf of YFD infected 5-year old Qinghuajiao trees grown in the experimental field of Fruit Research Institute of Chongqing Academy of Agricultural Sciences, at Diaojia town under natural conditions.

Recently, an emergent yellow-flower disease (YFD) was occurred in the green Chinese prickly ash (Qinghuajiao) trees in Chongqing, and showed a spread and dissemination tendency (Figure S1). The healthy Qinghuajiao trees showed green inflorescences, degenerate stamens, and flat leaves (Fig. 1a–c). However, the symptomatic Qinghuajiao trees showed yellow inflorescences, abortive pistils, intumescent and yellow stamens, and curled leaves (Fig. 1d–f). After an irreversible and degenerative progress, the diseased trees was died, and then causing severe yield reduction, and thus leading to huge economic losses.

### General view of the transcriptome results

**Figure 2:**
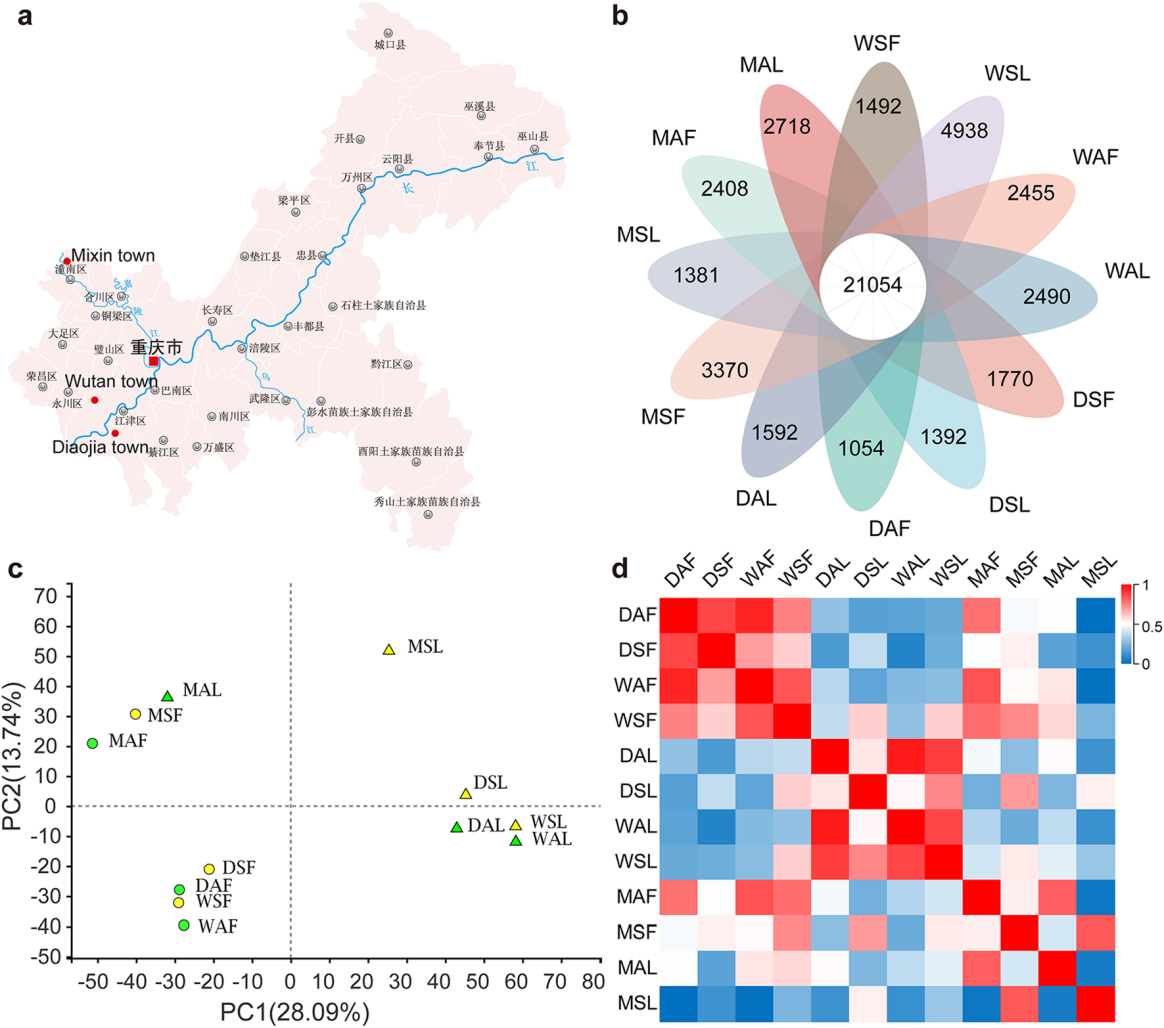
Overview of transcriptome results for 12 samples from three towns. (**a**) Distribution map of sampling points. Diaojia town, N29°12′03.25, E106°23′03.58; Mixin town, N30°22′35.19, E105°46′20.05; Wutan town, N29°26′05.51, E106°07′52.88. (**b**) Venn diagram of transcriptome results of 12 Qinghuajiao samples from three sampling points. (**c**) PCA analysis based on the gene expression profile of each sample. (**d**) Heat map shows the Pearson’s correlation of gene expression levels between samples. *DAF* Diaojia asymptomatic flower, *DSF* Diaojia symptomatic flower, *DAL* Diaojia asymptomatic leaf, *DSL* Diaojia symptomatic leaf, *MAF* Minxin asymptomatic flower, *MSF* Minxin symptomatic flower, *MAL* Minxin asymptomatic leaf, *MSL* Minxin symptomatic leaf, *WAF* Wutan asymptomatic flower, *WSF* Wutan symptomatic flower, *WAL* Wutan asymptomatic leaf, *WSL* Wutan symptomatic leaf.

To elucidate the molecular response for this YFD of Qinghuajiao, we collected the leaves and inflorescences of symptomatic and asymptomatic Qinghuajiao trees of the same variety from the experimental field of Fruit Research Institute of Chongqing Academy of Agricultural Sciences, in Diaojia Town, Wutan Town and Mixin Town at March 21, 2019 (Fig. 2a), and noted as DAF, Diaojia asymptomatic flower; DSF, symptomatic flower; DAL, Diaojia asymptomatic leaf; DSL, symptomatic leaf; and so on.

Through high-throughput sequencing RNA-Seq of the 12 samples of these three sample points, we obtained 80.45 Gb total clean data, and the raw and clean reads of all samples were close to each other (Table 1). By using Trinity software (https://github.com/trinityrnaseq/trinityrnaseq, Version v2.8.5), we assembled all clean data from scratch, and obtained 126,550 unigenes and 229,643 transcripts. The smallest and largest length were both 201 bp and 14,553 bp for the assembled unigenes and transcripts, and the N50 length was 1095 bp for unigenes, 1524 for transcripts (Table S1). And then the clean reads of each sample were compared with the reference sequence obtained by Trinity assembly, we obtained the mapping results of each sample, and we found a total of 21,054 genes that were detectable in all 12 samples (Fig. 2b and Table 1). Principal component analysis (PCA) showed that the 12 samples could be divided into four groups: DSF, DAF, WSF, WAF; DSL, WSL, WAL; MSF, MAF, MSL; and DAL, MAL, which was correlated to the sample location and tissue type, only DAL and MAL were separated from the groups that they were supposed to be in (Fig. 2c). Furthermore, the results of sample correlation matrix based on gene expression levels were consistent with the PCA results (Fig. 2d), which emphasized the reproducibility and reliability of our experimental samples.

**Table 1:** The statistical table of sequencing data.

Sample	Raw reads	Clean reads	Error rate (%)	Q20 (%)	Q30 (%)	GC content (%)	Mapped reads	Mapped ratio (%)
DAF	44,897,014	44,405,144	0.0253	97.92	93.77	44.46	35,720,896	80.44
DAL	46,698,684	46,102,750	0.0257	97.78	93.47	43.92	35,771,906	77.59
DSF	46,445,076	45,828,398	0.026	97.65	93.15	44.39	36,592,042	79.85
DSL	49,842,648	49,246,100	0.0256	97.81	93.51	44.36	39,092,396	79.38
MAF	45,373,972	44,875,490	0.0256	97.81	93.49	44.03	34,074,216	75.93
MAL	42,613,334	42,019,704	0.0259	97.71	93.28	44.33	31,259,428	74.39
MSH	46,484,696	45,746,648	0.0264	97.48	92.79	44.2	35,652,174	77.93
MSL	47,935,756	47,234,592	0.0259	97.7	93.28	44.13	37,091,480	78.53
WAF	45,745,828	45,253,928	0.0254	97.9	93.71	44.1	35,707,816	78.91
WAL	43,159,894	42,572,250	0.0255	97.84	93.63	44.17	32,649,024	76.69
WSF	44,532,338	44,001,784	0.0252	97.98	93.92	43.82	34,516,336	78.44
WSL	44,512,072	43,694,110	0.0253	97.91	93.82	44.09	35,206,070	80.57

### The total DEGs of asymptomatic and symptomatic Qinghuajiao trees

**Figure 3:**
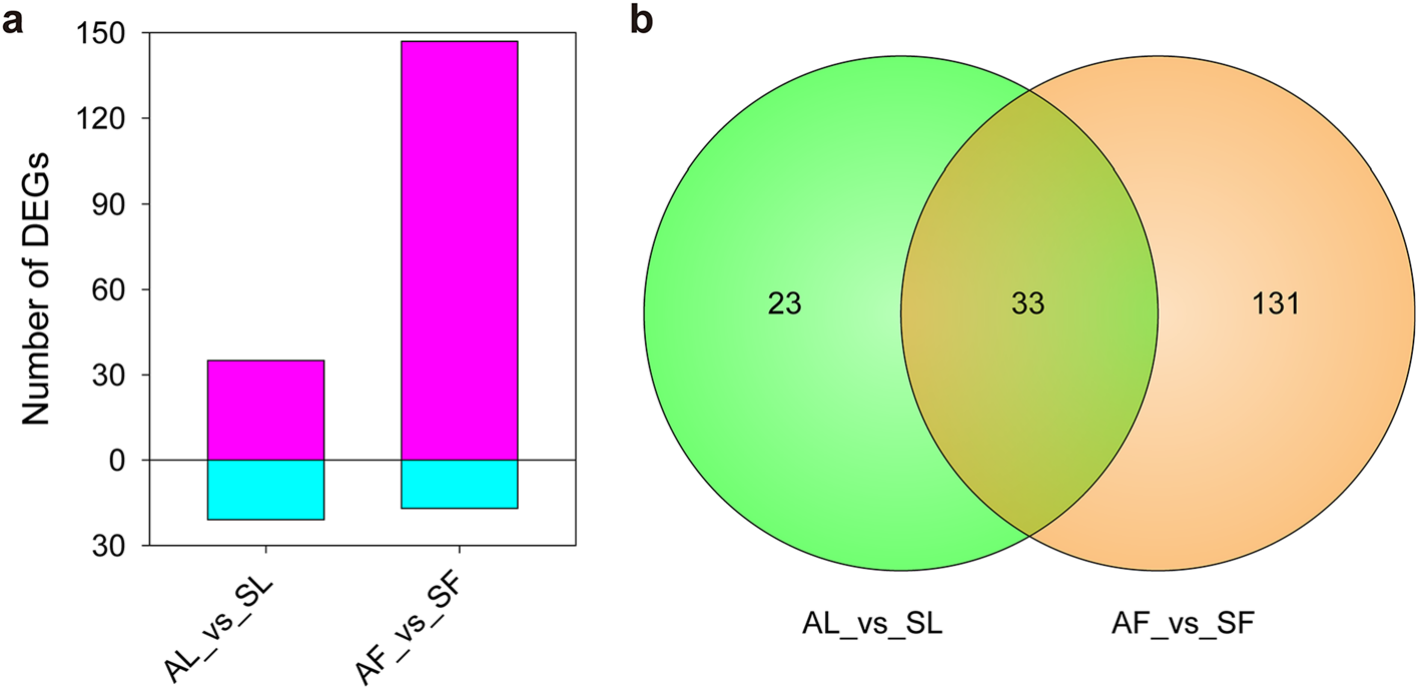
Up- and down- regulated genes in symptomatic Qinghuajiao trees. (**a**) Statistical results of up- and down- regulation genes in symptomatic Qinghuajiao trees. *AL* asymptomatic leaf, *SL* symptomatic leaf, *AF* asymptomatic flower, *SF* symptomatic flower. (**b**) A Venn diagram of differentially expressed genes (DEGs) between AL_vs_SL and AF_vs_SF groups. AL: DAL, WAL, MAL; SL: DSL, WSL, MSL; AF: DAF, WAF, MAF; SF: DSF, WSF, MSF. *DAF* Diaojia asymptomatic flower, *DSF* Diaojia symptomatic flower, *DAL* Diaojia asymptomatic leaf, *DSL* Diaojia symptomatic leaf, and so on.

According to the sample types, we divided the 12 samples into 4 groups: AF (DAF, MAF, WAF), SF (DSF, MSF, WSF), AL (DAL, MAL, WAL), and SL (DSL, MSL, WSL). In order to avoid the influence of environmental factors, the samples collected from three different towns were used as replicated samples. For instance, DAF, WAF, and MAF were used as replicates for AF samples. By using DESeq2 (http://bioconductor.org/packages/stats/bioc/DESeq2/, Version 1.24.0) software, we obtained 56 and 164 differentially expressed genes (DEGs, four fold expression difference with FDR (false discovery rate) value < 0.001) for AF_vs_SF and AL_vs_SL groups, respectively. There were 147 up-regulated and 17 down-regulated DEGs for AF_vs_SF group, and 35 up and 21 down DEGs for AL_vs_SL group, respectively (Fig. 3a, and Figs. S2, S3). Interestingly, we construct the diagram of Venn for these 2 gene sets, and, among the 220 DEGs, we found that only 33 DEGs were detected in both groups (Fig. 3b), which might be related to the YFD of Qinghuajiao.

### GO and KEGG analysis for DEGs

**Figure 4:**
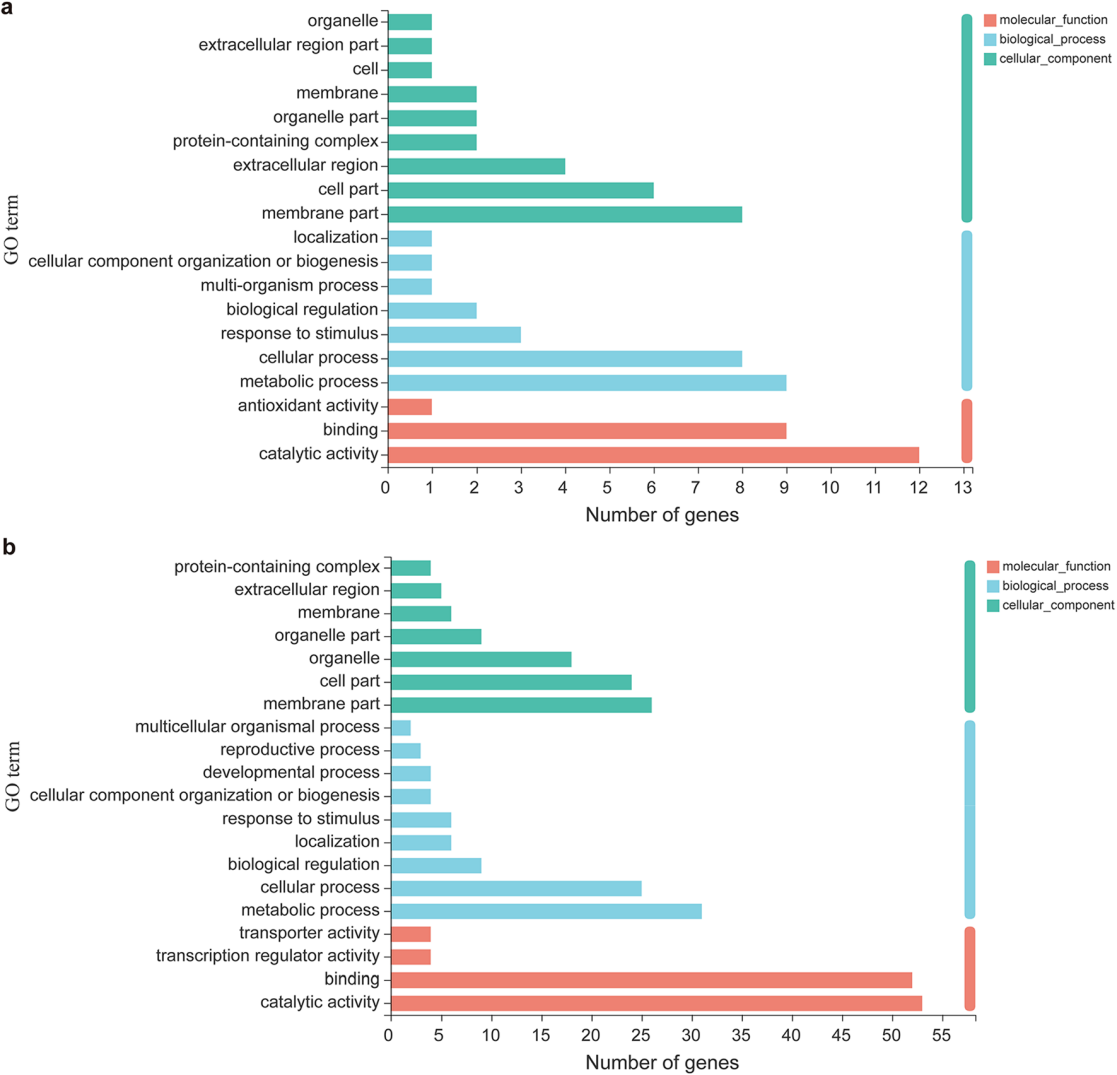
Gene ontology classification of DEGs for 2 groups. (**a**,**b**) GO classification of differentially expressed genes (DEGs) for AL_vs_SL (**a**) and AF_vs_SF (**b**) groups. AL: DAL, WAL, MAL; SL: DSL, WSL, MSL; AF: DAF, WAF, MAF; SF: DSF, WSF, MSF. *DAF* Diaojia asymptomatic flower, *DSF* Diaojia symptomatic flower, *DAL* Diaojia asymptomatic leaf, *DSL* Diaojia symptomatic leaf; and so on.

To better understand the functions of DEGs, we carried out Gene Ontology (GO) and Kyoto Encyclopedia of Genes and Genomes (KEGG) analysis by using the free online tools of Majorbio Cloud Platform (http://www.majorbio.com). For AL_vs_SL group, 22 DEGs were related to molecular function, in which “catalytic activity” (12) and “binding” (9) were the mainly GO terms; 25 DEGs touched upon biological process, and “metabolic process” (9) and “cellular process” (8) occupied a high percentage; 27 DEGs referred to cellular component, and “membrane part” (8) and “cell part” (6) made a major contribution to this category (Fig. 4a). In AF_vs_SF group, GO terms “catalytic activity” (53) and “binding” (52) also mainly contributed to the molecular function category (119); “catalytic activity” (31), “binding” (25), and “biological regulation” (9) were the major GO terms that related to biological process (90); GO terms “membrane part” (26), “cell part” (24), and “organelle” (18) occupied higher percentage in the cellular component category (Fig. 4b). In the KEGG annotation, 12 DEGs were divided into 10 categories for AL_vs_SL group, and 35 DEGs were referred to 24 categories for AF_vs_SF group (Table 2), among which, “Phenylpropanoid biosynthesis”, “Cyanoamino acid metabolism”, “Starch and sucrose metabolism”, “Amino sugar and nucleotide sugar metabolism”, “RNA transport”, and “Basal transcription factors” were shared for both groups. Interestingly, the “Phenylpropanoid biosynthesis” pathway has been reported to be involved in response to plant pathogen. Moreover, for AF_vs_SF group, 2 DEGs were related to the “Plant–pathogen interaction” pathway (Table 2), implying that this YFD diseases was possibly caused by plant pathogen.

**Table 2:** The statistical table of KEGG analysis.

Pathway ID	Description	AL_vs_SL_up	AL_vs_SL_down
map00196	Photosynthesis—antenna proteins	1	0
map00940	Phenylpropanoid biosynthesis	1	1
map00460	Cyanoamino acid metabolism	1	0
map00500	Starch and sucrose metabolism	1	1
map00030	Pentose phosphate pathway	0	1
map00520	Amino sugar and nucleotide sugar metabolism	0	1
map03013	RNA transport	1	0
map03022	Basal transcription factors	1	0
map04016	MAPK signaling pathway—plant	0	1
map04217	Necroptosis	0	1

Pathway ID	Description	AF_vs_SF_up	AF_vs_SF_down
map00310	Lysine degradation	0	2
map00260	Glycine, serine and threonine metabolism	1	0
map00410	beta-Alanine metabolism	1	0
map00940	Phenylpropanoid biosynthesis	6	0
map00945	Stilbenoid, diarylheptanoid and gingerol biosynthesis	1	0
map00460	Cyanoamino acid metabolism	1	0
map00960	Tropane, piperidine and pyridine alkaloid biosynthesis	1	1
map00052	Galactose metabolism	1	0
map00360	Phenylalanine metabolism	1	0
map00350	Tyrosine metabolism	1	1
map00130	Ubiquinone and other terpenoid-quinone biosynthesis	1	0
map00500	Starch and sucrose metabolism	1	0
map00230	Purine metabolism	1	0
map00520	Amino sugar and nucleotide sugar metabolism	1	0
map00240	Pyrimidine metabolism	1	0
map00950	Isoquinoline alkaloid biosynthesis	1	1
map03010	Ribosome	1	0
map03013	RNA transport	1	0
map03022	Basal transcription factors	1	0
map02010	ABC transporters	1	0
map04075	Plant hormone signal transduction	0	1
map04144	Endocytosis	2	0
map04626	Plant–pathogen interaction	2	0
map04712	Circadian rhythm—plant	0	1

*AL_vs_SL_up* the number of genes that up-regulated in symptomatic leaves, *AL_vs_SL_down* the number of genes that down-regulated in symptomatic leaves, *AF_vs_SF_up* the number of genes that up-regulated in symptomatic flowers, *AF_vs_SF_down* the number of genes that down-regulated in symptomatic flowers.

### The 2 groups shared 33 common DEGs

**Figure 5:**
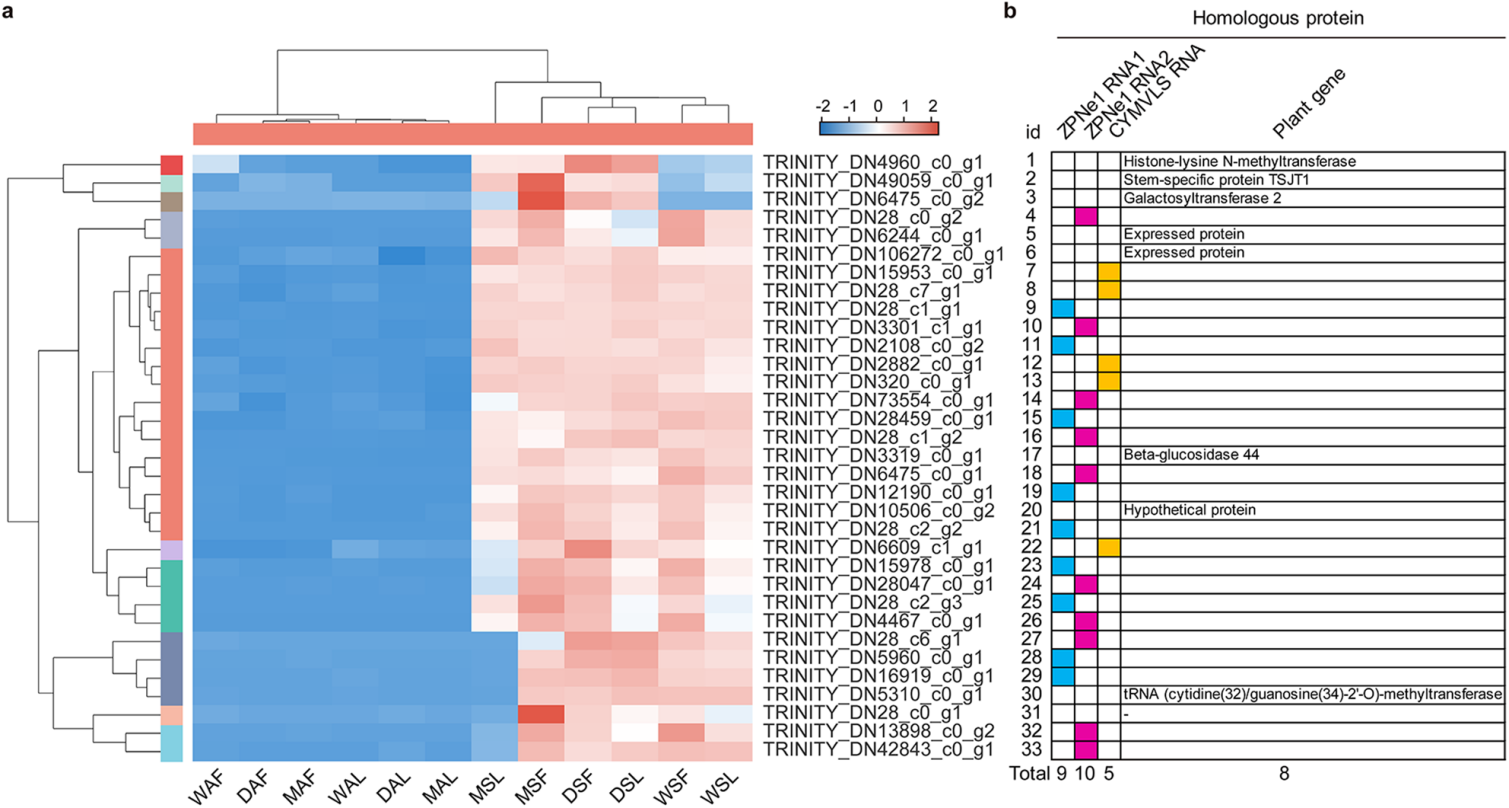
Expression and annotation of 33 common genes. (**a**) The expression heat map of 33 common genes in the 12 samples. *DAF* Diaojia asymptomatic flower, *DSF* Diaojia symptomatic flower, *DAL* Diaojia asymptomatic leaf, *DSL* Diaojia symptomatic leaf; and so on. Left panel (samples from asymptomatic trees), right panel (samples from symptomatic trees). (**b**) Blast results the 33 common genes. id 1–33 indicated the genes as shown in (**a**), from top to bottom, respectively. *ZPNe1 RNA1* Zhuye pepper nepovirus isolate ZPNe1 segment RNA1, *ZPNe1 RNA2* Zhuye pepper nepovirus isolate ZPNe1 segment RNA2, *CYMVLS RNA* Chicory yellow mottle virus large satellite RNA; Plant gene, homologous to expressed protein in other plant; –, no blast result.

**Figure 6:**
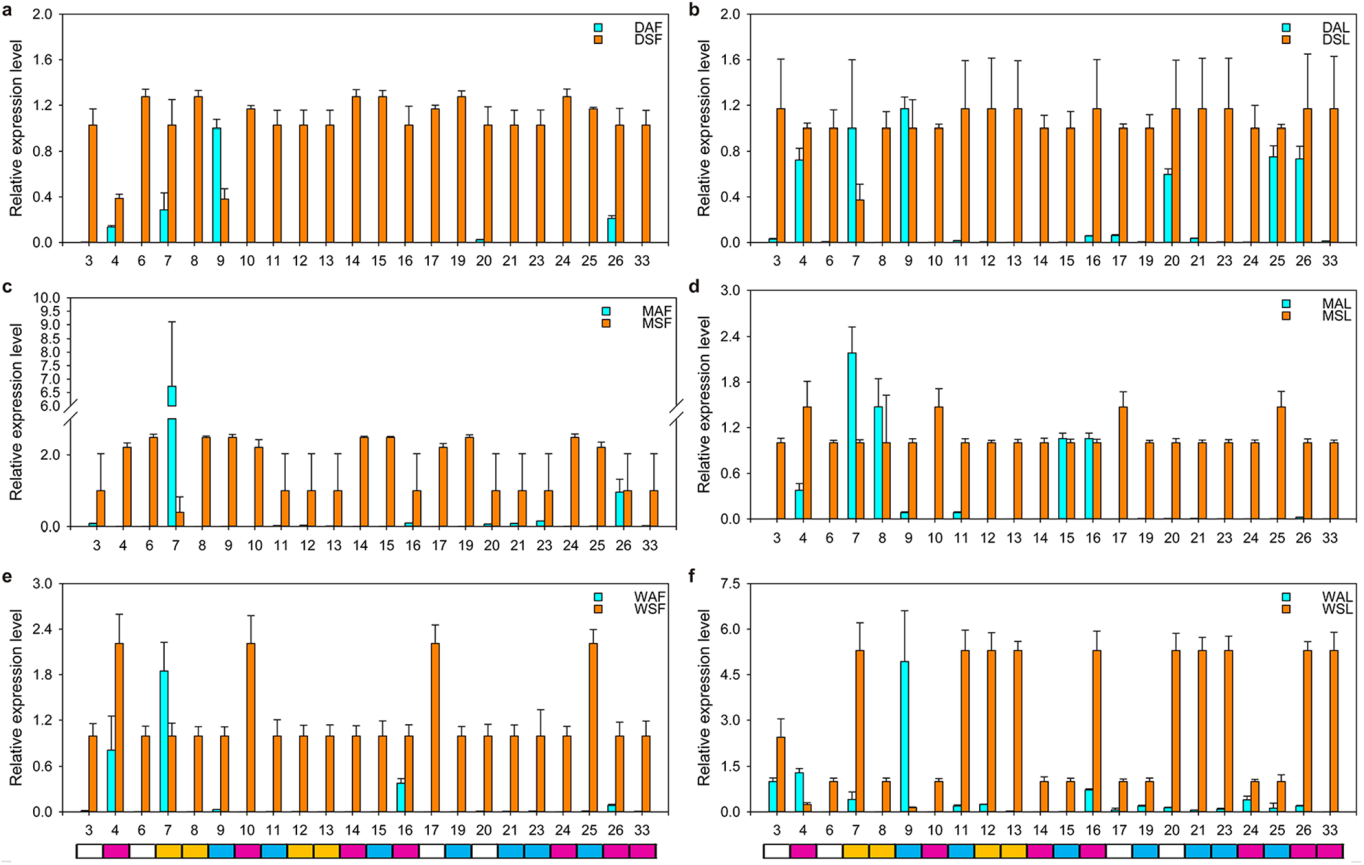
The expression level of 33 common genes detected by real-time qRT-PCR. (**a**–**f**) The normalized expression level of 33 common genes in DAF_vs_DSF (**a**), DAL_vs_DSL (**b**), WAF_vs_WSF (**c**), WAL_vs_WSL (**d**), MAF_vs_MSF (**e**), MAL_vs_MSL (**f**). The *Actin* for Qinghuajiao was used as internal control. The expression level of 9, 7, 26,16, 4, and 16 genes in samples from asymptomatic trees was set as 1 to figure (**a**–**f**), and then the expression levels of other genes was normalized to these for figure (**a**–**f**), respectively. *DAF* Diaojia asymptomatic flower, *DSF* Diaojia symptomatic flower, *DAL* Diaojia asymptomatic leaf, *DSL* Diaojia symptomatic leaf; and so on. 3, 4, 6, 7, 8, 9, 10, 11, 12, 13, 14, 15, 16, 17, 19, 20, 21, 23, 24, 25, 26, and 33 indicate TRINITY_DN6475_c0_g1 (Galactosyltransferase 2), TRINITY_DN28_c0_g2, TRINITY_DN106272_c0_g1 (Expressed protein), TRINITY_DN15953_c0_g1, TRINITY_DN28_c7_g1, TRINITY_DN28_c1_g1, TRINITY_DN3301_c1_g1, TRINITY_DN2108_c0_g2, TRINITY_DN2882_c0_g1, TRINITY_DN320_c0_g1, TRINITY_DN73554_c0_g1, TRINITY_DN28459_c0_g1, TRINITY_DN28_c1_g2, TRINITY_DN3319_c0_g1 (Beta-glucosidase 44), TRINITY_DN12190_c0_g1, TRINITY_DN10506_c0_g2 (Hypothetical protein), TRINITY_DN28_c2_g2, TRINITY_DN15978_c0_g1, TRINITY_DN28047_c0_g1, TRINITY_DN28_c2_g3, TRINITY_DN4467_c0_g1, and TRINITY_DN42843_c0_g1, respectively. The rectangles below the numbers represent: white, plant gene; purple, ZPNe1 RNA2; yellow, CYMVLS RNA; blue, ZPNe1 segment RNA1. ZPNe1 RNA1, Zhuye pepper nepovirus isolate ZPNe1 segment RNA1; ZPNe1 RNA2, Zhuye pepper nepovirus isolate ZPNe1 segment RNA2; CYMVLS RNA, Chicory yellow mottle virus large satellite RNA; Plant gene, homologous to expressed protein in other plant. Each analysis was repeated with three biological replicates. Error bars, ± SEM.

As shown in Fig. 3b, the AL_vs_SL and AF_vs_SF groups shared 33 common DEGs. More interestingly, most of the 33 common DEGs showed a consistent regulatory trend, that is to say, they were up-regulated in curled leaves and yellow flowers of the symptomatic trees (Fig. 5a), implying that these 33 DEGs might closely related to the YFD of Qinghuajiao. In addition, we further confirmed the expression level of these 33 DEGs in symptomatic trees by real time qRT PCR, the results were consistent with the transcriptome analysis (Fig. 6). Interestingly, the plant genes (NO.3, 6, 17, 20) were almost not expressed in the samples from asymptomatic trees, while were highly expressed in symptomatic trees, implying that these four genes might closely involve in the responses of Qinghuajiao to YFD. Almost the detected virus RNA homologous genes were all up-regulated in symptomatic trees. In addition, some virus RNA homologous genes were expressed in samples from healthy trees, hinting that some of the sampled healthy plants might be asymptomatic carrier.

### The blast results of the 33 common genes

To find out the proteins encoded by these 33 genes, we blasted these genes on the NCBI web site (https://blast.ncbi.nlm.nih.gov/Blast.cgi) by using the assembled sequences. Among the 33 genes, 9 genes are homologous to ZPNe1 RNA1 (Zhuye pepper nepovirus isolate ZPNe1 segment RNA1), 10 genes are homologous to ZPNe1 RNA2, 5 genes are homologous to CYMVLS RNA (Chicory yellow mottle virus large satellite RNA), which are all components of viruses; and 8 genes are homologous to other genes in plants (Fig. 5b). Interestingly, the 24 genes that homologous to virus RNA were all up-regulated in symptomatic plants (Figs. 5a and 6). Consider with the KEGG pathway enrichment results, it seemed that the YFD of Qinghuajiao might be caused by virus.

## Discussion

Several pathogens including fungi and phytoplasmas caused diseases have been reported in Chinese prickly ash. Infection of *Septoria pachyspora* results in leaf blotch in green Chinese prickly ash^14^. Aster yellows phytoplasma is associated with Prickly ash witches' Broom Disease in Korea^15^. Yang et al. reported that *Alternaria alternata* leads to brown leaf spot and panicle blight of *Zanthoxylum piperitum* in China^16^. *Alternaria alternate* also causes leaf spot on *Zanthoxylum dissitum* in China^17^. Little viral diseases have been reported in Chinese prickly ash. Green Sichuan pepper vein clearing-associated virus (GSPVCaV), a badnavirus, leads to vein clearing symptoms in green Chinese prickly ash^18^. These reports are all from the plant pathogen view to elaborate disease of Chinese prickly ash. In this study, we detected the transcriptome alteration of Qinghuajiao leaves and inflorescences that encountered the YFD disease, and obtained a total of 220 DEGs, which were involved in the molecular response of Qinghuajiao to this YFD. To our knowledge, this is the first transcriptome report in response to Qinghuajiao YFD.

The transcriptome profiling of cucumber leaves infected with powdery mildew (PM) revealed that the complex regulatory network for PM resistance involves plant hormone signal transduction, phenylpropanoid biosynthesis, plant–pathogen interaction and the MAPK signalling pathway^19^. Based on the KEGG pathway and GO enrichment of transcriptome data, Guo et al. showed a complex regulatory network for PM resistance in pumpkin that may also involve hormone signal transduction pathways^20^. Chen et al. reported that genes associated with plant hormone signal transduction, detoxification, phenylpropanoid biosynthesis, photosynthesis and chlorophyll metabolism were significantly affected by CMV infection in yellow passion fruit^21^. Transcriptome results showed that the moderate resistance of *Pinus pinaster* to *Fusarium circinatum* may be explained by the expression profiles pertaining to early recognition of the pathogen, the induction of pathogenesis-related proteins and the activation of complex phytohormone signaling pathways^22^. Some KEGG annotations of Qinghuajiao encountered with YFD were also belong to these previously reported to be response to plant–pathogen interaction by transcriptomic analysis. For instance, the “Plant–pathogen interaction” and “plant hormone signal transduction” pathways for AF_vs_SF group, and the “phenylpropanoid biosynthesis” for both groups. These results are important first step for insights on the molecular mechanism for the interaction between Qinghuajiao and YFD, and suggest that the phenylpropanoid biosynthesis and plant hormone signal transduction pathways were involved in the responses of Qinghuajiao to YFD.

Several virus diseases have been reported in plants. A subgroup B nepovirus, Potato virus B (PVB) infected the potatoes in central Peru, and leading to calico-symptom leaves^23^. The grapevines that infected by an *Enamovirus* member, GEV-1 (Grapevine enamovirus-1) exhibit redden or yellowing and downward rolling leaves^24^. Tomato black ring virus (TBRV), a typical member of *Nepovirus* genus within the *Secoviridae* family, infects a wide range of economically important plants worldwide, including tomato, strawberry, potato, celery, and artichoke^25^. Gaafar et al. reported that a caraway yellows virus (CawYV) infection results in systemic yellowing in Caraway^26^. Grapevine fan leaf virus (GFLV), a picorna-like plant virus, is severely in responsible for a widespread disease in vineyards worldwide^27^. The blast results of the up-regulated 30 common genes showed that most of these genes has highly homolog with virus RNA, implying that the YFD of Qinghuajiao might be related to virus. Further study will be conducted at the isolation of the virus that resulted in YFD of Qinghuajiao, the molecular character and the transmission routes of these viruses, so as to get the methods to suppress the transmission of these viruses, and thus to inhibit the further spread of YFD. In addition, although all most virus-infected plant diseases cause economic losses, Beaver-Kanuya and Harper had reported that four viruses infecting the pollen of Prunus species might have implications for biosecurity^28^. While the symptomatic Qinghuajiao trees have intumescent stamens, which could be used to improve male sterility in other crops.

Taken together, through high-throughput RNA-Seq analysis of 12 samples from three points, we revealed the molecular response of YFD of Qinghuajiao. We found a total 220 DEGs for the symptomatic trees. The Gene Ontology (GO) analysis showed that the major related GO terms of the DEGs were “catalytic activity” and “binding” to molecular function category, “metabolic process” (9) and “cellular process” to biological process category, and “membrane part” and “cell part” to cellular component category, respectively. The Kyoto Encyclopedia of Genes and Genomes (KEGG) results showed that the DEGs were divided into 10 categories for AL_vs_SL group and were referred to 24 categories for AF_vs_SF group. Particularly, the “phenylpropanoid biosynthesis” pathway that related to plant–pathogen interaction were found in both groups, and the “Plant–pathogen interaction” founded in AF_vs_SF group, implying that this Qinghuajiao YFD might cause by plant pathogen. Interestingly, we found 33 shared DEGs for the AL_vs_SL and AF_vs_SF groups, and most of them were all up-regulated in the samples from diseased trees. Among which, 24 genes were homologs to virus RNA, implying that this YFD of Qinghuajiao was related to virus. Although the mechanisms underlying the response of Qinghuajiao to YFD still require further research, the knowledge obtained from this study will serve as a useful genetic resource to facilitate further investigations of YFD in Qinghuajiao, and provide many possible directions for prevention and treatment of YFD of Qinghuajiao. Additionally, our study also provides a quick and preliminary approach to identify the causes of serious diseases occurring in plants with complex genomes under natural conditions.

## Materials and methods

### Materials

The green Chinese prickly ash (*Zanthoxylum schinifolium*, Qinghuajiao) was grown in Chongqing at natural conditions. The in inflorescences and leaves of asymptomatic and symptomatic Qinghuajiao trees of same variety (Qinghuajiao) were collected from the experimental field of Fruit Research Institute of Chongqing Academy of Agricultural Sciences, at Diaojia town (N29°12′03.25, E106°23′03.58), Mixin town (N30°22′35.19, E105°46′20.05), and Wutan town (N29°26′05.51, E106°07′52.88) in Chongqing as biological replicates to avoid environmental influences. The plants for photograph and samples collection were 5-year old, all leaves are fully developed antepenultimate leaves, and the flowers are shoot apical flowers. The experimental research on the plants described in this study comply with institutional, national and international guidelines.

### High-through put RNA-Seq

The inflorescence and leaf samples were collected at March 21, 2019, and was stored at − 80 °C at Biotechnology Research Center, Southwest University. The samples were then sent to Shanghai Majorbio Bio-pharm Technology Co., Ltd. to conduct High-through put RNA-Seq as previously described and with some modification^29^. Briefly, The mRNA was enriched using the oligo(dT) magnetic beads. The sample libraries were qualified and quantified by Agilent 2100 Bioanaylzer and Nanodrop2000. The library products were sequenced via Illumina Novaseq 6000. The raw data were filtered with the FASTQ_Quality_Filter tool from the FASTX-toolkit (http://hannonlab.cshl.edu/fastx_toolkit/, Version 0.0.14). And then using Trinity software (https://github.com/trinityrnaseq/trinityrnaseq, Version v2.8.5) to assemble all sample clean data from scratch^13^, and using TransRate (http://hibberdlab.com/transrate/, Version v1.0.3)^30^ and BUSCO (Benchmarking Universal Single-Copy Orthologs, http://busco.ezlab.org, Version 3.0.2)^31^ to Optimize and evaluate assembly. All assembled transcripts were compared with six major databases (NCBI_NR, NCBI non-redundant protein library, ftp://ftp.ncbi.nlm.nih.gov/blast/db/; Swiss-Prot, http://web.expasy.org/docs/swiss-prot_guideline.html; PFAM, http://pfam.xfam.org/; COG, Clusters of Orthologous Groups of proteins, http://www.ncbi.nlm.nih.gov/COG/; GO, Gene Ontology, http://www.geneontology.org; and KEGG, Kyoto Encyclopedia of Genes and Genomes, http://www.kegg.jp/kegg/kegg1.html, to obtain the annotated information for each transcript.

### Differential expression analysis

The transcriptome data was analyzed on the free online tools of Majorbio Cloud Platform (http://www.majorbio.com). The transcriptome was quantified by RSEM (http://deweylab.github.io/RSEM/, Version 1.3.1)^32^, and the amount of gene expression was expressed in the words TPM (Transcripts Per Million reads)^33^. The different expression gene (DEG) was analyzed by DESeq2 (http://bioconductor.org/packages/stats/bioc/DESeq2/, Version 1.24.0) as described by Love et al^34^. The significance threshold for the differential expression was FDR (false discovery rate) < 0.05 and a |log2 fold change | > 1. All DEGs were analyzed by Kyoto Encyclopedia of Genes and Genomes (KEGG) enrichment using KOBAS software (http://www.kegg.jp/kegg/kegg1.html)^35^. The corrected P-value is tested by Fisher's exact test, when the corrected P-value is < 0.05, significant enrichment of KEGG pathway is considered. The heat map was built by D3 (https://d3js.org/, version 7.0.0) with modification by Shanghai Majorbio Bio-pharm Technology Co., Ltd.

### RNA extraction and quantitative RT-PCR

Total RNA was extracted using the plant total RNA extraction kit (Tiangen, China). First-strand cDNA was synthesized from 1 µg total RNA using a reverse transcription kit with genomic DNA remover (Takara, Japan). Gene-specific primers are designed and listed in Table S2. Real-time PCR was performed on CFX96™ Optical Reaction Module (Bio-Rad, USA) using the Novostar-SYBR Supermix (Novoprotein, China) according to the manufacturer’s instructions. The *Actin* for Qinghuajiao was used as internal control. The normalized expression level were obtained by the Bio-rad CFX manager 3.1 software. Each analysis was repeated with three biological replicates.

### Ethics approval and consent to participate

The plant materials used in this study were collected from the experimental field of Fruit Research Institute of Chongqing Academy of Agricultural Sciences, and field permission was not necessary to collect the plant samples for this study. The authors declared that experimental research on the plants described in this study comply with institutional, national and international guidelines.

## Supplementary Information

Supplementary Information.

## Data Availability

The raw sequence data supporting the results of this article are available on the free online platform of Majorbio Cloud Platform (http://www.majorbio.com). Further information and requests for data and material should be directed to and will be fulfilled by Ming Luo (luo0424@126.com). The raw sequence data supporting the results of this article are also submitted to the Sequence Read Archive (SRA), and can be found by SRR14127436, SRR14127437, SRR14127438, SRR14127439, SRR14127440, SRR14127441, SRR14127442, SRR14127443, SRR14127444, SRR14127445, SRR14127446, and SRR14127447 in NCBI (https://www.ncbi.nlm.nih.gov/), and will be released at April 15, 2022.
