# Supplementary material for: Transcriptome analysis reveals the molecular mechanisms of response to an emergent yellow-flower disease in green Chinese prickly ash (Zanthoxylum schinifolium)
Source: Sci Rep. 2021 Sep 23;11:18886. doi: 10.1038/s41598-021-98427-5 (PMC8460732; doi:10.1038/s41598-021-98427-5)
Supplement: Supplementary file 1 — Supplementary Information. [file 41598_2021_98427_MOESM1_ESM.docx]

**Supplemental information**

## Supplementary Information

**Figure S1** Survey on the incidence of Yellow flower disease in main Qinghuajiao producing areas in China

**Figure S2** The expression heat map of differentially expressed genes in symptomatic leaves

**Figure S3** The expression heat map of differentially expressed genes in symptomatic leaves

**Table S1** Evaluation of assembly results

**Table S2** Primers used in real-time qRT-PCR

**Figure S1**


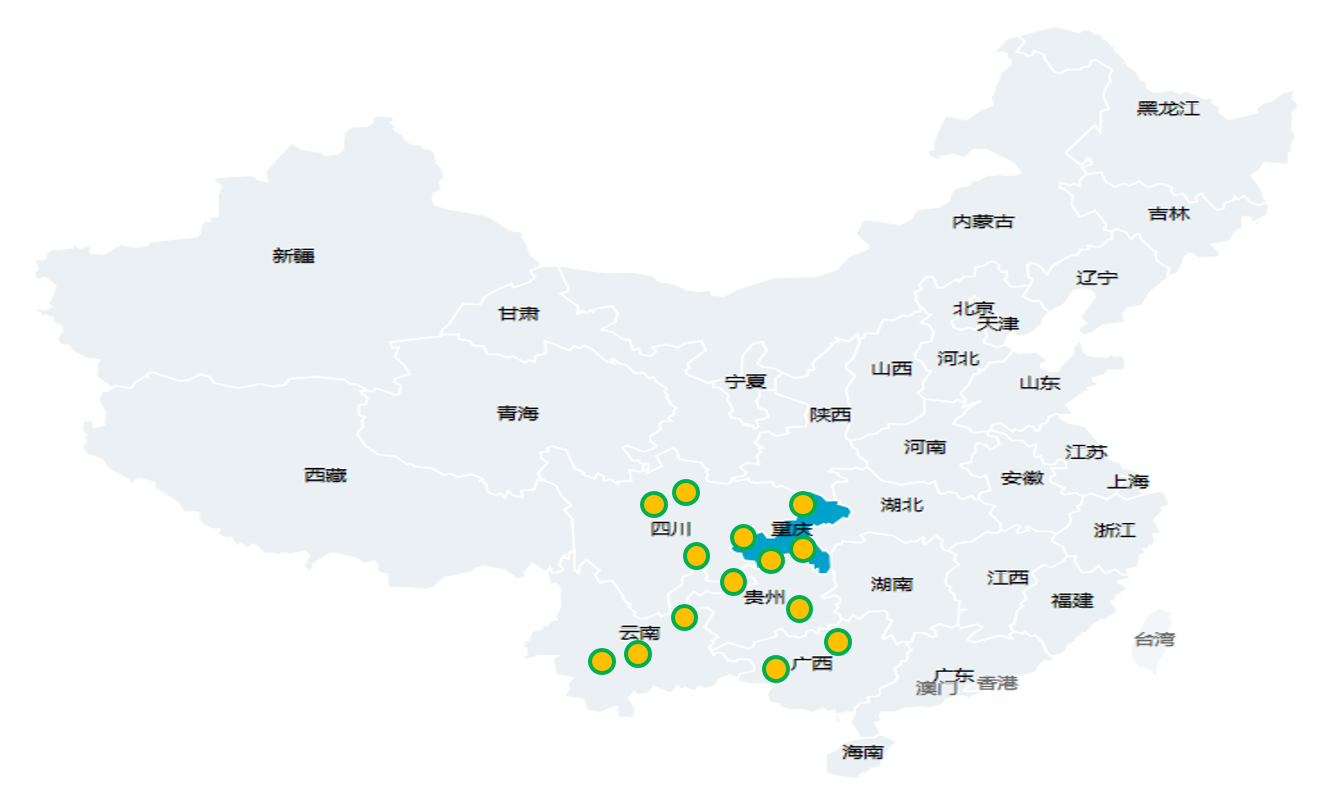


**Figure S1 Survey on the incidence of Yellow flower disease in main Qinghuajiao producing areas in China**

Survey areas: Jiangjin, Tongnan, Changshou, Fengdu, Fuling, Fengjie, and Jiulongpo in Chongqing municipality; Guang'an, Nanbu, Pingchang, Ziyang, and Deyang in Sichuan province; Baoshan, Shidian, and Zhaotong in Yunnan province; Zunyi and Bijie in Guizhou province; Baise and Nanning in Guangxi province.


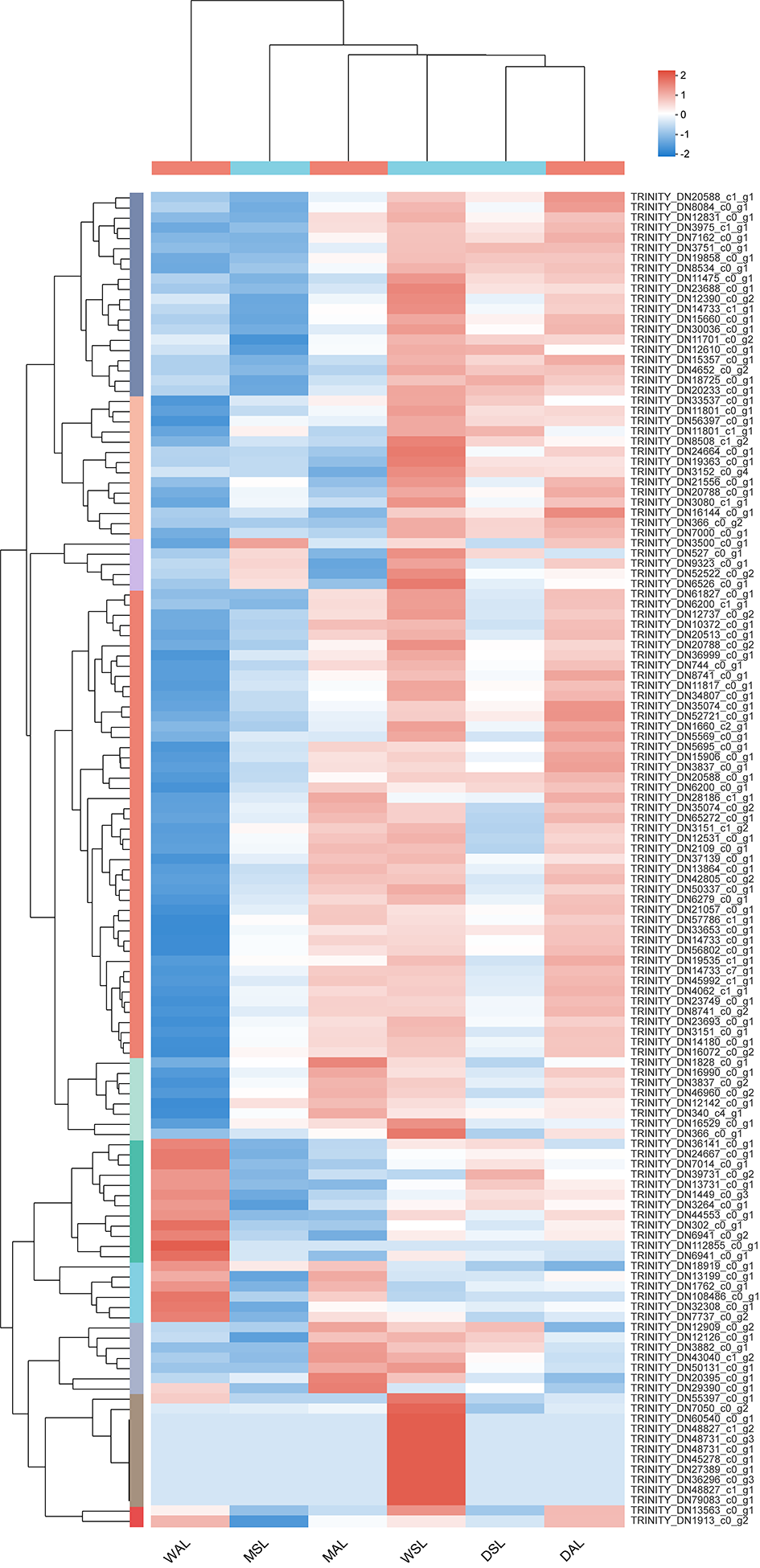


**Figure S2 The expression heat map of differentially expressed genes in** **symptomatic leaves.**

DAL, Diaojia asymptomatic leaf; DSL Diaojia symptomatic leaf; and so on.


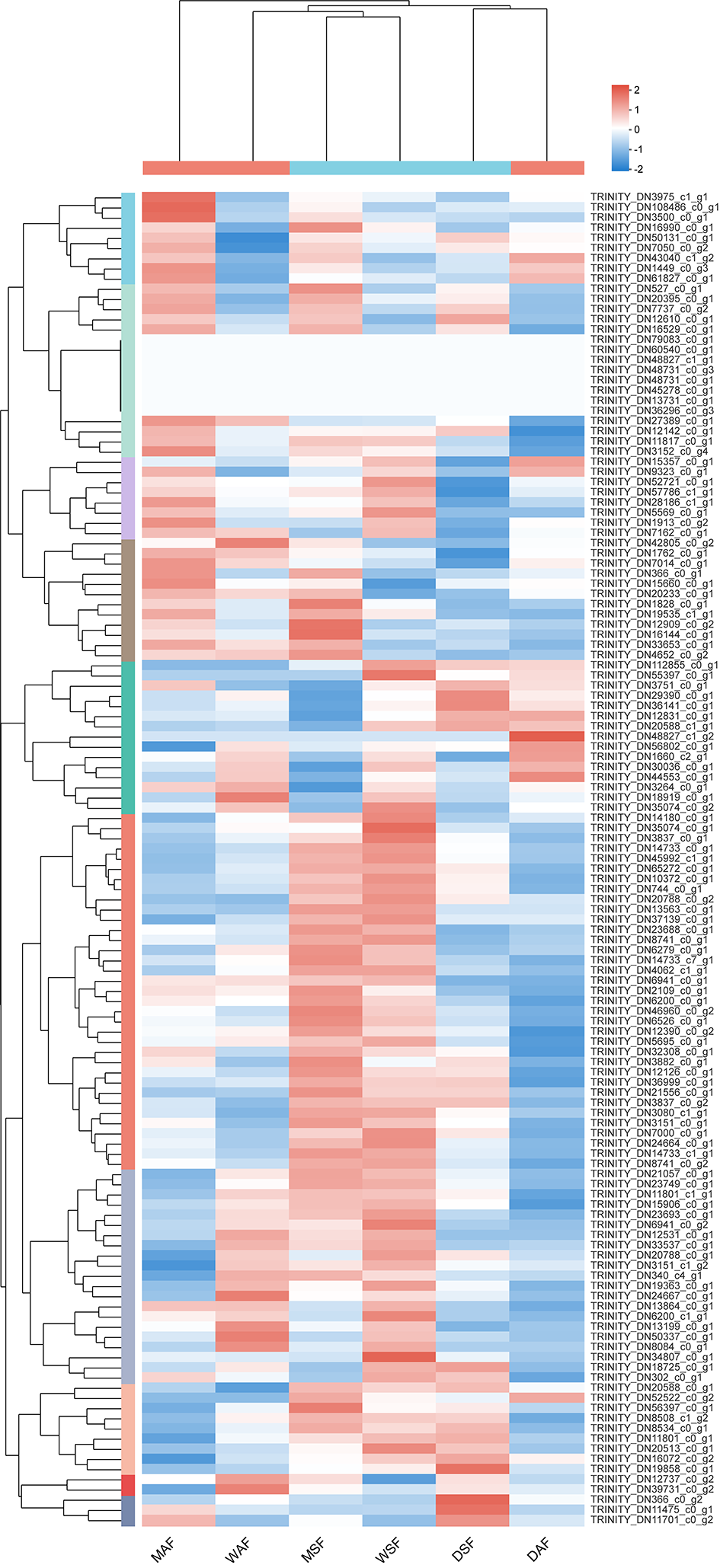


**Figure S3 The expression heat map of differentially expressed genes in** **symptomatic leaves.**

DAF, Diaojia asymptomatic flower; DSF Diaojia symptomatic flower; and so on.

**Table S1 Evaluation of assembly results**

| Type | Unigene | Transcript |
| --- | --- | --- |
| Total number | 126550 | 229643 |
| Total base | 92871164 | 211928700 |
| Largest length (bp) | 14553 | 14553 |
| Smallest length (bp) | 201 | 201 |
| Average length (bp) | 733.87 | 922.86 |
| N50 length (bp) | 1095 | 1524 |
| E90N50 length (bp) | 2359 | 2058 |
| Fragment mapped percent (%) | 52.01 | 75.945 |
| GC percent (%) | 39.84 | 39.94 |
| TransRate score | 0.21935 | 0.33232 |
| BUSCO score | C:75.2%[S:72.4%;D:2.8%] | C:75.2%[S:72.4%;D:2.8%] |

**Table S2 Primers used in real-time qRT-PCR**

| Gene | Forward (5'-3') | Reverse (5'-3') |
| --- | --- | --- |
| TRINITY_DN28_c1_g2 | TCTACGAGGTTAGAGCCGGT | TCATCGCAGTGCCCGTAAAA |
| TRINITY_DN3301_c1_g1 | CTTCCGCGTTTCTCGAAGGT | ATTAAAGCGCACGGATAGGC |
| TRINITY_DN28_c1_g1 | CCCAAAGGTACGGTAGCTGG | GGCAGCAGCCTTAATGGGTA |
| TRINITY_DN3319_c0_g1 | GGGATTGCTTCAGCTCCACT | ATCGGGAAGGACCAGCTACT |
| TRINITY_DN5310_c0_g1 | ACGTGGTTTGGCTTCCTCAA | GCCCACTCGAATGTGGATGA |
| TRINITY_DN320_c0_g1 | ATTGCTGCTAGACGCCATGT | GGCTGACCGGATAGTCATCG |
| TRINITY_DN12190_c0_g1 | TTGGGCATGCAAACCACATT | TGAATCCGATGGCGTGCATA |
| TRINITY_DN2108_c0_g2 | TGCCGCAGAGCAAGATTGTA | GTCCAGAACACGTGGGGAAA |
| TRINITY_DN15978_c0_g1 | AGCCTCCGTTGGTTTAGGTG | TTTGGCCCTCCCTGCTAAAG |
| TRINITY_DN73554_c0_g1 | TGTCGGAGGGAGATACCCAG | AGATTTCAAGGCGCTCGGAT |
| TRINITY_DN28_c7_g1 | TCCACCTCATCGAGGCTACT | TGCGCGAACATCTAGAACCT |
| TRINITY_DN42843_c0_g1 | GCATGGGCGAATGTGTAAGC | ACTACCTTCGAGAAACGCGG |
| TRINITY_DN15953_c0_g1 | GGCTGGTGCCATCCTACAAT | GCAACACGGTCCATACGTTC |
| TRINITY_DN28047_c0_g1 | CACCTAAACCAACGGAGGCT | CCATCCCTTAGTGGGAGGGA |
| TRINITY_DN106272_c0_g1 | AGTCCTCAATGGACAGCTCG | CTTGAGGTTCGACCGTCGTTA |
| TRINITY_DN2882_c0_g1 | TTCGACATGGCGTCTAGCAG | AACTGTCCTTCGCTTCTCGG |
| TRINITY_DN10506_c0_g2 | ACGTACGGTAGGGTTCCAGA | CACACAACATCAAGCACGCA |
| TRINITY_DN6475_c0_g1 | AATGAGAGCTTGCGGTGTCA | ATGATCCGAACTGAGCCCAC |
| TRINITY_DN4467_c0_g1 | AAGCTCGATACTTGGCGAGG | AAGGGCTCAACAGAAGGTCG |
| TRINITY_DN5960_c0_g1 | ATGGCCTAACTGTTCACGGA | AGGGGATCTGCCCATTAAAGC |
| TRINITY_DN28459_c0_g1 | GCTGTGCCTGGACCATTCTT | AAAGCATTCCCGCCATCCAA |
| TRINITY_DN28_c0_g2 | ATTGGTGGTCTGAAAGCCGT | TCAATCGGGAGGAAAACCCC |
| TRINITY_DN28_c2_g3 | CTCCCACCTAAACCAACGGA | CCCCTCCACCTTCCCATAAG |
| TRINITY_DN16919_c0_g1 | AGTCACTCCCCACTCACTCA | TCACAGTGCATCCCGATTGT |
| TRINITY_DN28_c6_g1 | TCATTCCTTCCTCGCGTTGT | TTTGCGGACAACTCACCGTA |
| TRINITY_DN28_c2_g2 | TCAACCGCTTCGAGATTGGC | GCTGTCTTGCTCTCCGATCA |
| TRINITY_DN28_c0_g1 | CCATTGGCACTCCAACTCTGA | AAGTCGGTGAGACCACCTGT |

**Abbreviations**

COG, Clusters of Orthologous Groups of proteins; GO, Gene Ontology; KEGG, Kyoto Encyclopedia of Genes and Genomes; YFD, yellow-flower disease; DEGs, different expressed genes; PCA, principal component analysis ZPNe1 RNA1, Zhuye pepper nepovirus isolate ZPNe1 segment RNA1; ZPNe1 RNA2, Zhuye pepper nepovirus isolate ZPNe1 segment RNA2; CYMVLS RNA, Chicory yellow mottle virus large satellite RNA
